# Human serum-derived hydroxy long-chain fatty acids exhibit anti-inflammatory and anti-proliferative activity

**DOI:** 10.1186/1756-9966-30-59

**Published:** 2011-05-17

**Authors:** Shawn A Ritchie, Dushmanthi Jayasinghe, Gerald F Davies, Pearson Ahiahonu, Hong Ma, Dayan B Goodenowe

**Affiliations:** 1 Phenomenome Discoveries, Inc. Saskatoon, Saskatchewan, Canada; 2 University of Saskatchewan, Saskatoon, Saskatchewan, Canada

**Keywords:** Long-chain fatty acid, colorectal cancer, aging, screening, inflammation, NFκB

## Abstract

**Background:**

Circulating levels of novel long-chain hydroxy fatty acids (called GTAs) were recently discovered in the serum of healthy subjects which were shown to be reduced in subjects with colorectal cancer (CRC), independent of tumor burden or disease stage. The levels of GTAs were subsequently observed to exhibit an inverse association with age in the general population. The current work investigates the biological activity of these fatty acids by evaluating the effects of enriched human serum extracts on cell growth and inflammation.

**Methods:**

GTAs were extracted from commercially available bulk human serum and then chromatographically separated into enriched (GTA-positive) and depleted (GTA-negative) fractions. SW620, MCF7 and LPS stimulated RAW264.7 cells were treated with various concentrations of the GTA-positive and GTA-negative extracts, and the effects on cell growth and inflammation determined.

**Results:**

Enriched fractions resulted in poly-ADP ribose polymerase (PARP) cleavage, suppression of NFκB, induction of IκBα, and reduction in NOS2 mRNA transcript levels. In RAW264.7 mouse macrophage cells, incubation with enriched fractions prior to treatment with LPS blocked the induction of several pro-inflammatory markers including nitric oxide, TNFα, IL-1β, NOS2 and COX2.

**Conclusions:**

Our results show that human serum extracts enriched with endogenous long-chain hydroxy fatty acids possess anti-inflammatory and anti-proliferative activity. These findings support a hypothesis that the reduction of these metabolites with age may result in a compromised ability to defend against uncontrolled cell growth and inflammation, and could therefore represent a significant risk for the development of CRC.

## Background

Fatty acid metabolism is intricately linked to the regulation of inflammatory processes, which underlie numerous diseases including cancer. For example, arachidonic, decosahexanoic and eicosapentanoic acids (AA, DHA and EPA) can be metabolized into both pro-inflammatory prostaglandins and leukotrienes, as well as into inflammation-resolving lipoxins, protectins and resolvins [1-3]. The failure to resolve acute inflammation through a lack of conversion to these latter products can result in a chronic inflammatory state, which over time can drive the development of inflammation-associated conditions including cancer, neurodegeneration, and others [4-10]. Functionally, many of these lipids have been shown to mediate their inflammation-associated effects through pathways involving the transcription factor NFκB and subsequent downstream pro-inflammatory molecules such as TNFα, IL-1β, COX2, and NOS2, for example [11-16].

Recently we reported on a novel class of hydroxylated long-chain fatty acids (called GTAs for gastrointestinal tract acids) present in the serum of healthy subjects and significantly reduced from the serum of colorectal cancer (CRC) patients [17,18]. Structurally, the molecules resemble very long chain (28 carbon) mimetics of the resolvins and protectins, containing multiple double bonds and at least two hydroxyl groups. The levels of GTAs do not change following treatment and show no correlation with tumor stage, suggesting that the reduction is not caused by the presence of the disease [17,18]. An inverse association between GTAs and age in the average-risk population further suggests that the reduction exists prior to cancer development, and may therefore represent a causal factor for the establishment and/or progression of the disease [18]. However, little is currently known about the biochemical role these molecules play in the disease process. The work reported herein, therefore, was carried out to investigate the effects of GTAs *in vitro *through the treatment of various cell lines with semi-purified GTA-enriched human serum extracts.

## Methods

### Cell lines and tissue culture

SW620, MCF-7 and RAW264.7 were purchased from ATCC and cultured in high glucose DMEM, 10% FBS at 37°C, 5% CO_2_. Cells were seeded at 1 × 10^6^/well in 6-well plates 24 hours prior to treatment with varying concentrations of GTA+ve extract, GTA-ve extract or vehicle (DMSO). RAW264.7 cells were pretreated with the extracts for 4 hours followed by the addition of LPS at 1 ug/ml (cat. No. L4391, Sigma) for 20 hours. Cells were harvested using a 2:1 ratio of Versene and TryPLe express (Gibco). The cell pellet was washed twice with phosphate buffered saline (PBS) and the stored at -80°C until extracted. Cell photographs were taken at 200× magnification on a phase-contrast EVOS digital microscope. All experiments were performed at least three times in duplicate or triplicate wells.

### Serum extraction, chromatography and mass spectrometry

Commercially available lyopholized human serum (Randox Laboratories, Canada) was resolubilized in double de-ionized water. The serum was extracted with 1:5 ratio of 1% ammonium hydroxide:ethyl acetate (Commercial grade, VWR) as previously described [17]. Ethyl acetate extracts were evaporated to dryness under reduced pressure (37°C/100 rpm) and re-suspended in methanol. Reverse phase silica (15 - 20 mg; WP C18 silica, 45 μm, 275 Å) was added into the serum methanol extract and evaporated to complete dryness under reduced pressure (45°C/150 rpm), which was then subjected to reverse phase flash column chromatography (FCC) with a step gradient elution; acetonitrile - water 25:75 to 100% acetonitrile. Eluent was fractionated into 12 aliquots (F1 - F12), which were each analysed for GTA content using HPLC-coupled tandem mass spectrometry on an ABI QSTAR XL mass spectrometer as previously described [17].

### Proliferation assays

Cell proliferation was determined using the MTT assay (3-(4,5-dimethylthiazol-2-yl)-2,5-diphenyltetrazolium bromide). Cell suspensions were prepared at a concentration of approximately 10^5 ^cells per ml as determined by standard hemocytometry, and cultured in 6-well multi-well plates. Prior to MTT analysis, cells were sub-cultured in phenol red-free DMEM medium to avoid interference with the colorimetric analysis of the purple formazan MTT product. Following treatment with serum extracts, cells were treated with MTT followed by washing with PBS, DMSO solubilization of the formazan product, and subjected to spectrophotometric analysis at 570 nm.

### Protein analysis

Cell pellets were resuspended in ice-cold lysis buffer (20 mM Tris (pH 7.5), 150 mM NaCl, 0.5 mM EDTA, 0.1 mM EGTA, 0.1% NP-40 plus 1X mammalian cell anti-protease cocktail (Sigma)). The cells were lysed using multiple freeze-thaw cycles followed by pulse sonication on ice and centrifugation at 3000 rpm for 5 minutes at 4°C to remove cell debris. Western blot analysis of these protein lysates was performed as previously described [19]. Briefly, equivalent amounts of protein were assessed by Bradford protein assay using BioRad Protein Reagent and resolved by 10% sodium dodecyl sulfate-polyacrylamide gel electrophoresis (SDS-PAGE). Following electrophoresis the proteins were trans-blotted onto nitrocellulose membranes (Pall-VWR). The membranes were blocked overnight at 4°C on a gyratory plate with 5% molecular grade skim milk powder (BioRad Laboratories) in phosphate-buffered saline (PBS) containing 0.1% Tween-20 (PBST). Primary and secondary antibody incubations and subsequent washes were carried out in the same buffer. Primary antibodies were obtained from Santa Cruz Biotechnology. The primary antibody for GAPDH was purchased from Sigma. Secondary HRP antibodies were purchased from BioRad. Blots were immunoprobed overnight with primary antibodies at a 1:1000 dilution. Secondary HRP antibody was applied at room temperature on a gyratory plate at a concentration of 1:10,000 for 30 min. Following multiple washes, an enhanced chemiluminescence detection system (Dupont-NEN) was used to detect the target antigen/antibody complexes. Blots were then stripped at 50°C for 30 minutes in a Tris-buffered 20% SDS/1% 2-mercaptoethanol stripping solution, washed and re-probed with GAPDH antibody (Sigma) to verify protein loading equivalency. For ELISA analysis, raw cells were treated as described above and conditioned medium or cell lysates were used to determine concentrations of TNFα (Cat. No. KMC3011, Invitrogen), and IL-1β (Cat. No. MLB00B, Quantikine), according to the manufacturer's instructions.

### Nitric oxide assay

Nitrite concentration in conditioned media was measured by Griess Reagent (Cat. No. G2930, Promega) according to the manufacturer's instructions.

### Quantitative Real-Time PCR

Total RNA was isolated from cell pellets using Trizol (Cat. No.15596-018, Invitrogen) as per manufacturer's instruction. RNA was resuspended in 50 μL of DEPC treated water and stored at -80°C. RNA concentration and purity was determined by spectrophotometry at 260 and 280 nm. Reverse transcription was performed using qScript cDNA super mix (Cat No. 95048-100, Quanta Biosciences). PCR was conducted by using Fast SYBR Green Master Mix (Cat No. 4385612, AB Applied Biosystems) on an Applied Biosystems Step One Plus Real-time PCR system. The relative number of each transcript copy was normalized by house-keeping gene Beta Actin. Real-time PCR primers used were as follows: NOS2 forward, CACCTTGGAGTTCACCCAGT; NOS2 reverse, ACCACTCGTACTTGGGATGC; COX2 forward, CCCCCACAGTCAAAGACACT; COX2 reverse, CTCATCACCCCACTCAGGAT; TNFα forward, AGAAGTTCCCAAATGGCCTC; TNFα reverse, GTCTTTGAGATCCATGCCGT; IL-1β forward, TGTGAAATGCCACCTTTTGA; IL-1β reverse, TGAGTGATACTGCCTGCCTG.

### Clinical Samples

Serum from a previously reported CRC patient and control population originating from Chiba University was equally pooled [17]. Ethyl-acetate extracts of the pooled control and CRC serum were subject to HPLC-coupled tandem mass spectrometry to determine relative GTA levels as previously described [17].

### Statistical Methods

Where data is averaged, error bars represent 1 standard deviation (S.D.) of the mean. Significance was determined if p < 0.05 using unpaired Student's T test (Microsoft Excel).

## Results

### Treatment of cells with un-enriched human serum extracts

**Figure 1:**
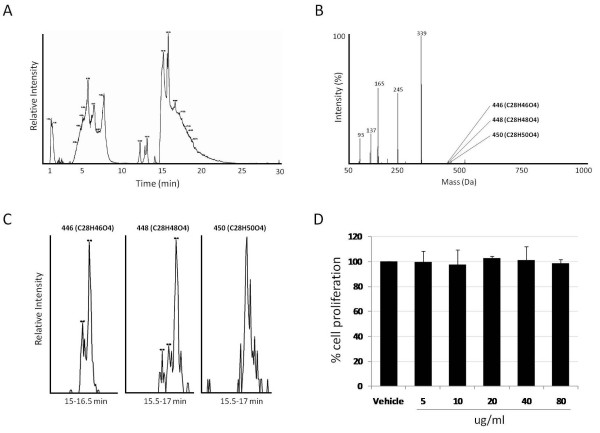
**Total ion chromatogram of crude serum organic extract**. (A) Total ion current of bulk serum following liquid/liquid extraction and HPLC-coupled mass spectrometry as explained in the methods. (B) Extracted mass spectra of all masses from (A). (C) Extracted ion chromatograms of GTAs 446, 448 and 450 from the total ion current shown in A. (D) Cell proliferation, as assayed by MTT, for SW620 cells treated with up to 80 ug/ml of the crude serum extract.

We first determined whether crude serum ethyl acetate extract, prior to chromatographic enrichment of GTAs, would have any effect on cellular growth by treating cells with commercially available bulk human serum extracts (see methods). The total ion chromatogram (TIC) of the organic fraction following HPLC-coupled time-of-flight (TOF) mass spectrometry is shown in Figure 1A. The extracted mass spectra of the complete TIC is shown in Figure 1B, which was dominated by various free fatty acids but contained detectable levels of GTAs including those with masses of 446 (C28H46O4), 448 (C28H48O4) and 450 (C28H50O4) Da (Figures 1B and 1C). By calculating the peak areas of the three chromatograms, we estimated that these three GTAs represented no more than 0.15% of the total ion current in the sample. Incubation of SW620 cells with up to 80 ug/ml of the extract for 48 hours showed no effect on cell proliferation (Figure 1D) or any effects on cell morphology as assessed by light microscopy (not shown).

**Figure 2:**
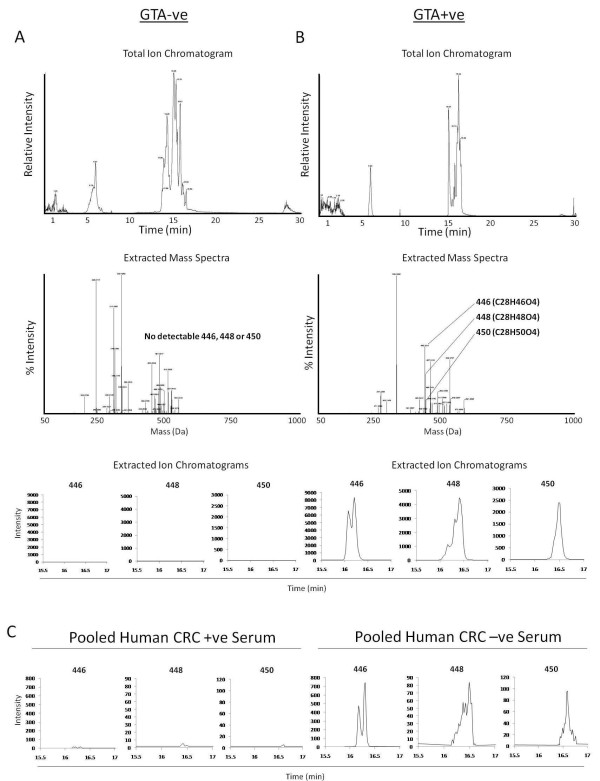
**Mass spectrometry characterization of semi-purified GTA-ve and GTA+ve extracts**. (A) Crude serum extract (as shown in Figure 1) was subject to flash column chromatography as described in the methods resulting in two adjacent eluates, one positive and one negative for the presence of GTAs. The total ion chromatogram (top), extracted mass spectra (middle), and extracted ion chromatograms for three GTAs (GTA446, 448 and 450; bottom) of the GTA-ve fraction. (B) Same as (A) for the GTA+ve fraction. (C) For comparison, the extracted ion chromatograms of GTA446, 448 and 450 from the extracts of serum pooled from 20 CRC patients and 20 controls is shown.

Organic serum extract was next subjected to flash column chromatography as described in the methods, resulting in 12 fractions which were subsequently analyzed by HPLC-MS to determine GTA content. Although other components were present in all the fractions, only fraction 9 out of the 12 was enriched for the C28 GTAs (referred to as the GTA+ve fraction). A GTA negative control fraction (fraction 8, lacking any detectable GTAs) was also selected for the studies described below. Representative total ion chromatograms, extracted mass spectra and selected ion chromatograms of the three C28 GTAs for the GTA-ve and GTA+ve fractions are shown in Figures 2A and 2B, respectively. By comparing the sums of the selected ion chromatograms of the three GTAs to the total ion currents, we estimated that the GTA+ve fraction contained approximately 21% C28 GTAs while the GTA-ve fraction had no detectable levels (bottom panel of Figures 2A and 2B). The non-GTA background components for both fractions were similar, and the most abundant non-GTA components in the GTA+ve fraction were also the most abundant components in the GTA-ve fraction. Therefore, the two fractions were compositionally similar other than the 21% GTA content of the GTA+ve fraction, which represented an approximately 143-fold enrichment of the three C28 GTA metabolites over the crude organic serum extract (as shown in Figure 1A). These fractionations were repeated several times with consistent results. We therefore concluded that the fractions were sufficiently matched for investigating biological activity as described below. For comparison, the relative levels of the three C28 GTAs from 40 pooled CRC patients' serum and serum from 40 matched control subjects is shown in Figure 2C.

### GTA+ve human serum extracts inhibit cell proliferation and induce PARP fragmentation

**Figure 3:**
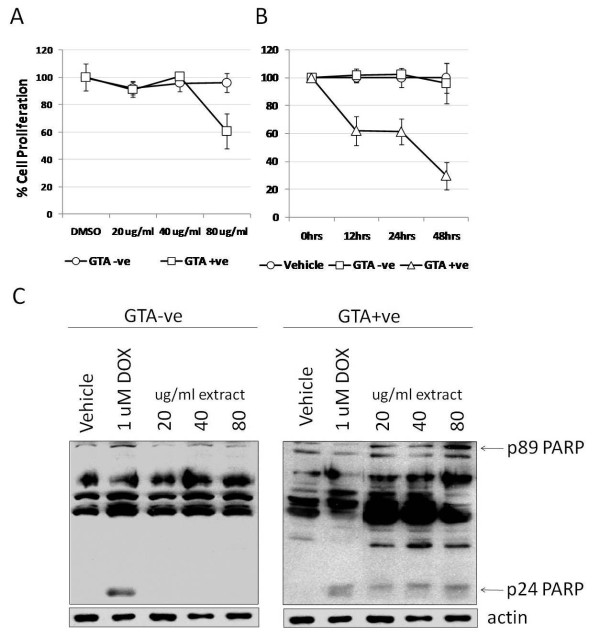
**Proliferation of SW620 cells treated with GTA+ve and GTA-ve extracts**. (A) SW620 cells were incubated with increasing concentrations of GTA+ve and GTA-ve extracts for 24 hours and proliferation assayed by MTT. (B) The 80 ug/ml concentration of GTA+ve and GTA-ve extracts was then used to treat cells for up to 48 hours and the effect on cell proliferation assayed by MTT. Data are expressed as percent of vehicle or 0 hrs ± 1S.D. (C) Representative Western blot analysis of caspase-mediated PARP cleavage fragments resulting from treatment with GTA+ve and -ve extracts. Experiments were repeated at least three times.

We determined whether the enriched GTA+ve fraction had any effects on cell viability compared to the GTA-ve fraction by treating SW620 cells for 24 hrs with three concentrations of each fraction and measuring the effect on cell proliferation by MTT assay. The GTA+ve fraction showed a 40% reduction in cell viability at a dose of 80 ug/ml (Figure 3A) while GTA-ve treatment had no effect. Treatment up to 48 hrs using 80 ug/ml showed the same 40% reduction as early as 12 hrs, which dropped further to 70% by 48 hrs (Figure 3B). No effect on cell proliferation was observed with the GTA-ve fraction or vehicle (DMSO). Evidence of apoptotic activity was determined by the detection of poly(ADP-ribose) polymerase (PARP) cleavage products through Western blot (Figure 3C). A number of PARP cleavage products including the hallmark 89 and 24 kDa fragments, as well as others (Figure 3C), were induced following 48 hrs treatment with GTA+ve fraction, but not with GTA-ve treatment, suggesting a possible pro-apoptotic function of GTAs.

**Figure 4:**
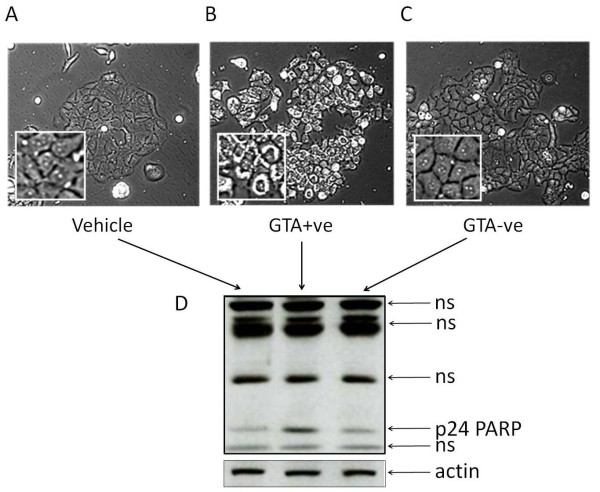
**Treatment of MCF7 cells with GTA+ve and GTA-ve extracts**. MCF7 cells were incubated with vehicle (A), 80 ug/ml GTA+ve extract (B), and 80 ug/ml GTA-ve extract (C) and cells photographed using phase-contrast light microscopy (200×). (D) Western analysis of PARP cleavage products; ns, non-specific.

We repeated the studies in MCF7 cells, which upon treatment with GTA+ve fraction showed gross cellular changes visible through phase-contrast microscopy including the appearance of apoptosomes and enlarged nuclei that were not observed with vehicle or GTA-ve treatments (Figures 4A, B and 4C). GTA+ve treatment in MCF-7 cells also resulted in the exclusive induction of the 24 kDa PARP cleavage product relative to vehicle or GTA-ve treatment (Figure 4D), further suggesting a pro-apoptotic activity of GTA-containing extracts.

### GTA+ve extracts inhibit pro-inflammatory markers

**Figure 5:**
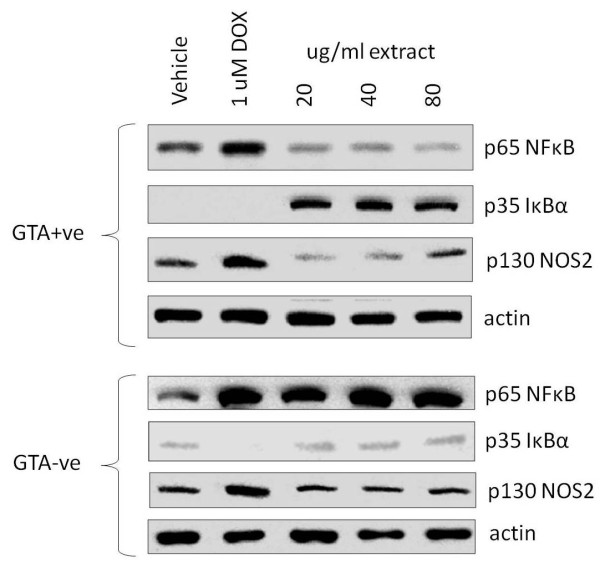
**Western analysis of NFκB, IκBα and NOS2 in SW620 cells treated with three concentrations of GTA+ve and GTA-ve extracts and doxorubicin (DOX)**. Representative Western blots showing protein levels of NFκB, IκBα and NOS2 in SW620 cells treated with GTA+ve and GTA-ve extracts (see methods).

The structural resemblance of GTAs to the inflammation-resolving protectins and resolvins prompted us to investigate the effect of GTA+ve extract on pro-inflammatory markers. Treatment of SW620 cells for 24 hours resulted in a profound inhibition of NFκB protein level with as little as 20 ug/ml GTA+ve extract, accompanied by an equally profound induction of IκBα, neither of which was observed with GTA-ve extract (Figure 5). Protein levels of nitric oxide synthase (NOS2) were also inhibited in cells treated with the GTA+ve fraction (particularly 20 and 40 ug/ml), but not in cells treated with the GTA-ve fraction (Figure 5).

**Figure 6:**
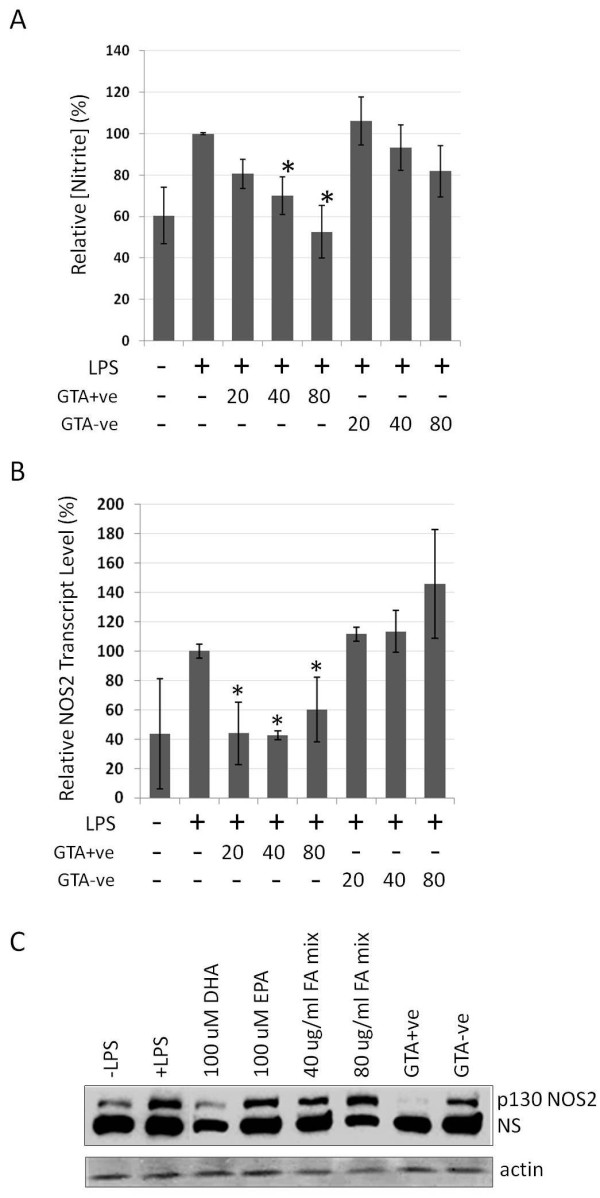
**Determination of nitric oxide status in RAW264.7 cells treated with GTA+ve and GTA-ve extracts**. RAW264.7 cells were pre-treated for 4 hours with GTA+ve or GTA-ve extracts followed by the addition of LPS (1 ug/ml) for 20 hours. (A) Nitric oxide levels in cells were determined using Griess reagent, (B) NOS2 mRNA transcript levels were determined by real-time rtPCR, and (C) NOS protein (treatment with 80 ug/ml) assessed by Western blot (NS, non-specific). Asterisks indicate p < 0.05 relative to LPS treatment alone, and FA mix in (C) represents a 100 uM equal mixture of 18:1, 18:2 and 18:3 fatty acids. Data are expressed as the average of three duplicate experiments ± 1S.D.

To explore further the effect of GTAs on modulating inflammation, we employed the RAW264.7 mouse macrophage line in which a pro-inflammatory state can be induced by treatment with lipopolysaccharide (LPS). RAW264.7 cells were treated for 4 hours with GTA+ve and GTA-ve fractions prior to the addition of LPS, and the effects on various proinflammatory markers evaluated. We observed no affect on RAW264.7 cell growth or proliferation rates during the 20 hours post-GTA treatment. RAW264.7 cells treated with GTA+ve fractions prior to LPS stimulation showed a significant dose-dependent reduction (p < 0.05) in the generation of nitric oxide as assessed through the production of nitrite using the Griess reagent system (Figure 6A), which was mirrored by low levels of NOS2 mRNA transcripts (Figure 6B) and protein levels (Figure 6C). For comparison (and as controls), cells were also treated with various combinations of free fatty acids including EPA, DHA and equimolar mixtures of 18:1, 18:2 and 18:3 (FA mix), of which only 100 uM DHA showed any protective effect on NOS2 protein induction (Figure 6C).

**Figure 7:**
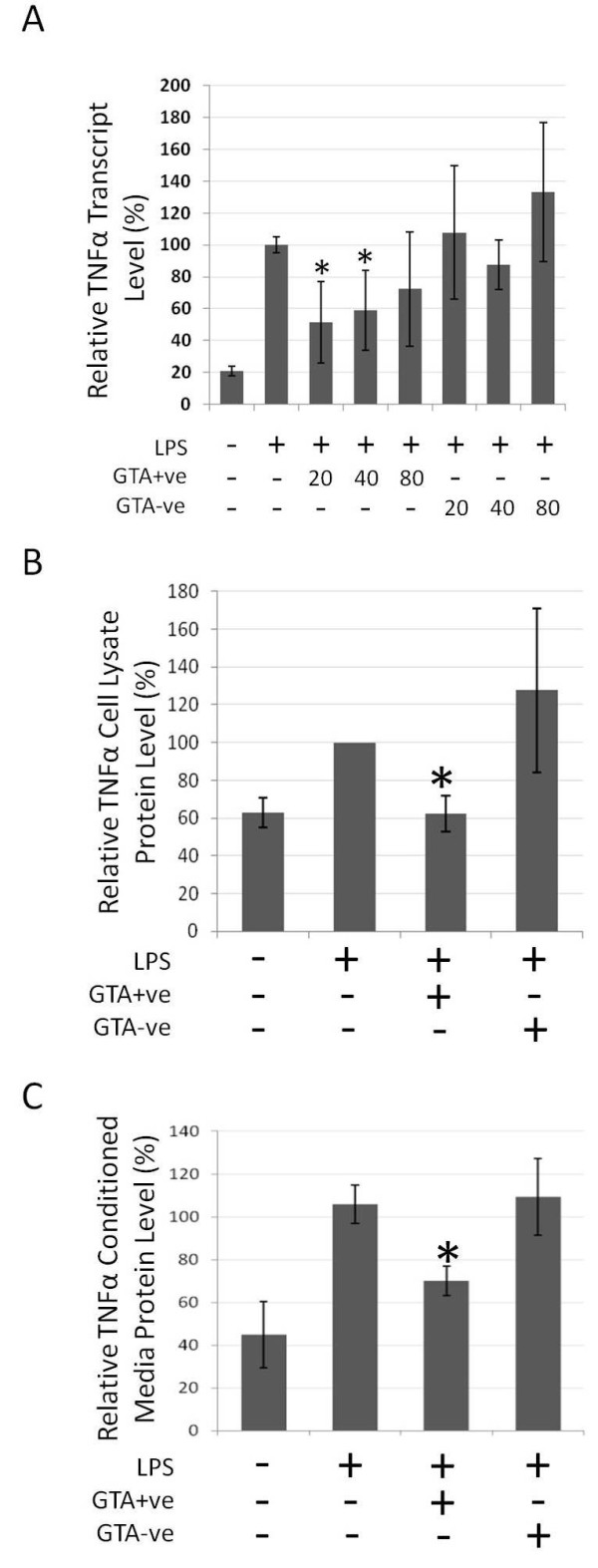
**TNFα response in RAW264.7 cells treated with GTA+ve and GTA-ve extracts**. RAW264.7 cells were pre-treated for 4 hours with GTA+ve or GTA-ve extracts followed by the addition of LPS (1 ug/ml) for 20 hours. (A) TNFα mRNA transcripts as determined by real-time rtPCR, (B) TNFα relative protein levels in cell lysates following 80 ug/ml treatment, and (C) TNFα protein levels in conditioned media as determined by ELISA. Asterisks indicate p < 0.05 relative to LPS treatment alone. Data are expressed as the average of three duplicate experiments ± 1S.D.

**Figure 8:**
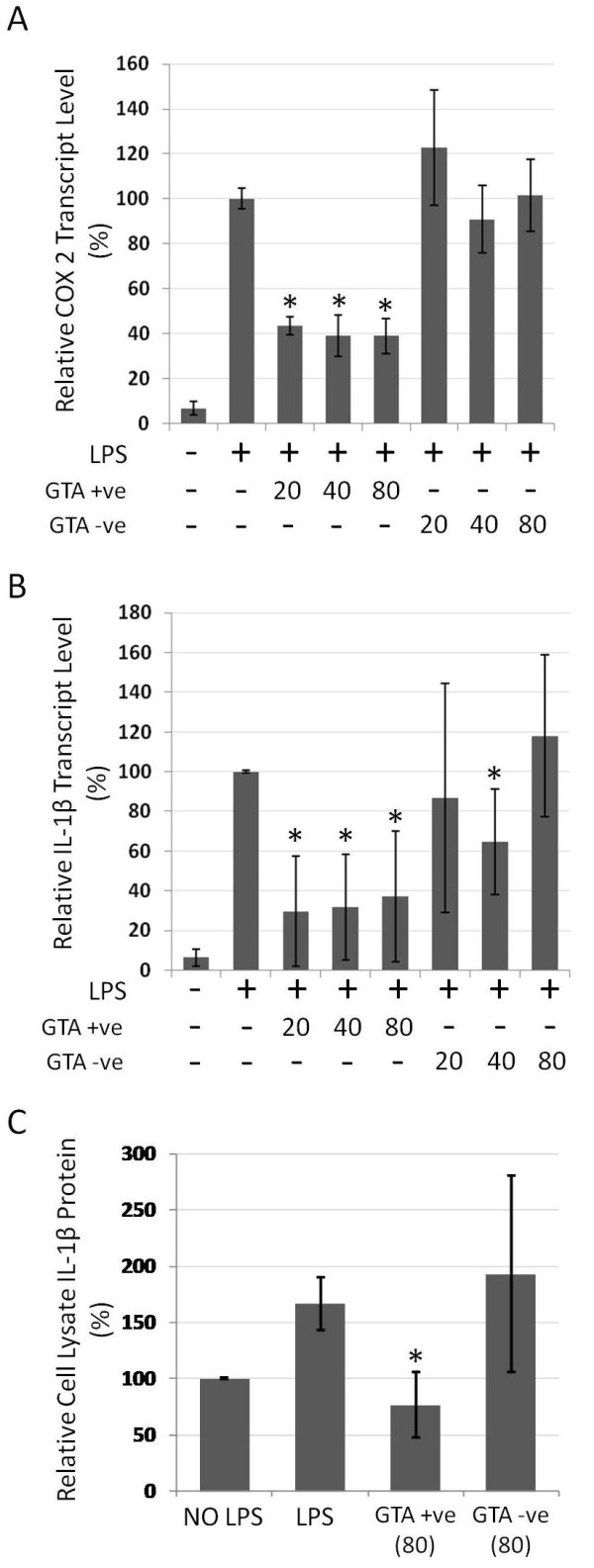
**COX2 and IL-1β response in RAW264.7 cells treated with GTA+ve and GTA-ve extracts**. RAW264.7 cells were pre-treated for 4 hours with GTA+ve or GTA-ve extracts followed by the addition of LPS (1 ug/ml) for 20 hours. (A) COX2 and (B) IL-1β mRNA levels were determined by real-time rtPCR. (C) IL-1β levels following 80 ug/ml treatment in cell lysates as determined by ELISA. Asterisks indicate p < 0.05 relative to LPS treatment alone. Data are expressed as the average of three duplicate experiments ± 1S.D.

Similar effects were observed with TNFα upon treatment with GTA+ve extract, which showed significantly reduced mRNA transcript levels (p < 0.05, Figure 7A) as well as protein levels in cell lysates and conditioned media (Figures 7B and 7C, respectively). Consistent with the above findings, transcript levels of COX2 and IL-1β (Figures 8A and 8B), as well as IL-1β protein levels (Figure 8C), were also significantly reduced (p < 0.05) with GTA+ve treatment. The results indicate that human blood extracts containing GTAs have anti-proliferative and anti-inflammatory properties that GTA-ve extracts lack.

## Discussion

The regulation of inflammation and the ability to control cell growth are two processes intricately linked with cancer. When acute inflammatory processes are not resolved by the appropriate enzymatic conversion of fatty acid mediators into specific oxygenated products [1,20,21], a state of chronic inflammation can ensue, which can further lead to sporadic DNA mutations, the activation of pro-oncogenic pathways and ultimately cancer (for example see [22]). When such detriments occur, they normally trigger a cascade of intracellular events leading to the induction of apoptotic-mediated cell death. Thus it is the fine control between inflammatory and apoptotic processes, likely early in life, which might be a key determinant of one's risk of subsequent cancer development.

Based on the tumor-independent reduction of GTAs previously reported in CRC patient serum [17], their age-related reduction in the general population [18], and their structural resemblance to the inflammation-resolving protectins and resolvins, we hypothesized that GTAs might represent a novel endogenous cancer-protective metabolic system. Although we focused specifically on a subset of 28-carbon GTAs, the GTA family comprises a large number of structurally related novel hydroxylated polyunsaturated ultra long-chain fatty acids ranging in size between 446 and 596 Da and containing up to 36 carbons [17].

In studies completed to date, GTAs appear to represent a human-specific metabolic system as they have only been detected in human serum (or plasma) and not in the serum or plasma of other mammals including mice, rats, cows, dogs, and rabbits. Likewise, GTAs are absent from several types of plant-based products such as grains and seed oils, as well as human tissues including colonic tumors and normal colon epithelium (unpublished observations). This human exclusivity has lead us to speculate that the gut microbiota, in combination with currently unknown dietary precursors, may be involved in their catabolism. The results shown here represent the first report of GTA biological activity, which revealed that cells treated with GTA+ve extracts had reduced proliferative capacity coinciding with PARP fragmentation, significantly down-regulated NFκB expression, increased IκBα levels, and numerous down-regulated inflammatory markers including nitric oxide, NOS2, IL-1β, TNFα and COX2. Given the critical role of NFκB in regulating both apoptosis and inflammation and its association with aging, our data suggests that the protective effects of GTAs are mediated, at least in part, through NFκB signalling. A reduction of GTAs over time could therefore be involved in compromising one's ability to protect against chronic inflammation and possibly cancer.

### GTAs, fatty acids, and proliferation

Our observation that GTA+ve extracts dose-dependently reduce cell proliferation, accompanied by the appearance of multiple PARP cleavage products with different molecular weights in SW620 cells but only the 24 kDa fragment in MCF-7 cells, suggests a complex cell-specific interplay between different proteases. Although it has been reported that caspase-3 activation can result in the 89 and 24 kDa fragments and that cathepsin-b and granzyme-b can produce fragments of 50 and 64 kDa, respectively [23], further work will be required to investigate GTA-specific protease activation. Our evidence of apoptosis upon treatment with GTAs is consistent with numerous other reports showing pro-apoptotic effects mediated through polyunsaturated long chain fatty acids (PUFAs). For example, docosahexanaeoic acid (DHA) has been shown to promote apoptosis through numerous pathways including cytochrome-c mediated caspase activation [24,25], inhibition of the regulatory subunit of PI3-kinase, and reduction of PTEN phosphorylation [24,26]. Others have shown that DHA and the PUFA punicic acid ultimately exert their intrinsic effects through dissipation of the mitochondrial membrane potential [27,28], and that DHA and butyrate can promote apoptosis by altering mitochondrial Ca^2+ ^levels [29]. Treatment of various cell lines, for example LAPC-4 prostate cancer-derived cells, with PUFAs, has been shown to reduce proliferation and induce apoptosis [30]. There are also studies demonstrating the inhibitory effects of omega-3 PUFAs on growth and angiogenesis of chemically induced as well as transplanted tumor model systems [31-33]. The observation of reduced cell growth in the presence of GTA+ve extract is therefore consistent with a large body of literature showing similar effects with exposure to long-chain PUFAs (see [34] for review).

In addition to its anti-proliferative effect, GTA+ve extract also protected against the LPS-mediated induction of several pro-inflammatory proteins including TNFα, IL-1β, NOS2 and COX2, and inhibited the production of nitric oxide. Central to these two effects (reduced proliferation *and *inflammation) was the concomitant inhibition of NFκB upon GTA+ve treatment in SW620 colonic epithelial cells, which correlated precisely with increased levels of the inhibitory protein IκBα, likely due to stabilization stemming from compromised ubiquitin-dependent proteosomal targeting [35]. The inhibition of NFκB is relevant to *both *apoptotic processes and inflammation, as discussed further below.

### NFκB and cell proliferation

NFκB, a transcription factor represented by a series of subunits harbouring discrete DNA binding and transactivational functionality, is implicated in both intrinsic and extrinsic apoptotic pathways (see [36] for review) and has been shown to prevent apoptosis as well as promote transformation in epithelial-derived cancers [37]. Mechanistically, in the absence of NFκB signalling, inhibitor-of-apoptosis proteins (IAPs) fail to suppress assembly of the death-inducing complex II, which allows for the TRADD-mediated activation of caspase-8 and subsequent apoptosis [36,38]. Furthermore, IAPs can directly promote the ubiquitin-mediated degradation of the NFκB-inducing serine/threonine kinase (NIK), ultimately resulting in NFκB activation [39]. Although a detailed discussion on this topic is out of scope, it is well established that activated NFκB is associated with an anti-apoptotic pro-survival advantage which is relevant given our data showing that GTA+ve extracts reduced NFκB expression. These observations are consistent with the reported biological activity of the resolvins and protectins, which have been shown to exert both pro-apoptotic effects [40] and the resolution of inflammation by attenuating cytokine levels in an NFκB-dependent manner [41]. One limitation of our study was that we were unable to determine NFκB levels in RAW264.7 cells, which will require further investigation upon the generation of sufficient quantities of either enriched extract, or more preferably, purified synthetic GTAs. However, the dramatic reduction of NFκB upon GTA treatment in colon tumor cells is highly relevant given the reduced levels of circulating GTAs in CRC patients [17,18] and the well-established inflammatory component of this disease [42].

### NFκB and inflammation

Besides its anti-apoptotic role, NFκB represents a key link between inflammation and cancer (see [43] for review), and in particular, is considered a master regulator of intestinal immunological function and activator of factors involved in driving intestinal inflammation [44-46]. NFκB activation has been observed in numerous GI-related conditions including inflammatory bowel disease [47], Crohn's disease [48], ulcerative colitis [35], inflamed intestinal mucosa [49] as well as CRC [50-53]. It has been shown that the NFκB transcriptional activity in gastric mucosa is induced during aging [53], that positive NFκB expression as assessed through immunohistochemistry is observed in 73.5% of human CRC tumors independent of age [50], and that levels of NFκB, IKKα and COX2 in tumor epithelial cells from CRC patients are significantly higher than adjacent normal tissue [52].

Also relevant to NFκB activation and intestinal inflammation is the reported differential regulation of Toll-like receptors (TLRs) by fatty acids with differing saturation states [54-56]. TLRs are single membrane-spanning proteins involved in the recognition of microbial-derived molecules harbouring pathogen-associated molecular patterns (PAMPs) and activation of various immune cell responses [57]. Upon activation, TLRs activate NFκB though a complex signalling network culminating with the activation of IKKα, followed by ubiquitination and subsequent degradation of IκBα and translocation of the P65 Rel A domain to the nucleus where it binds to and activates the expression of numerous pro-inflammatory genes [44]. Specifically, TLR4 has been shown to confer responsiveness to a number of lipids including lipid A, the primary biologically active component of LPS, and that in contrast to saturated fatty acids similar to those found in lipid A, unsaturated fatty acids inhibit NFκB activity through inhibition of TLR4 or other TLR-associated molecules [55,56]. Therefore, it is possible that the anti-inflammatory effects associated with GTAs are being exerted through inhibition of TLRs upstream of NFκB. Further work to investigate this hypothesis is warranted.

### NFκB, aging and gut microbiota

A final point concerning NFκB and GTA metabolism is that both have age-related implications. We previously showed that in the general population, there is an inverse association between the circulating levels of GTA-446 and age, and that the rate of decline correlated precisely with the increase in CRC incidence with age [18]. As for NFκB, there is substantial literature regarding its various age-related facets [53,58-63]. For example, it has been shown that NFκB protein levels, as well as several NFκB-targeted pro-inflammatory cytokines, are elevated in endothelial cells of old versus young subjects, which were also accompanied by decreased IκBα levels in the older group [60]. Microarray analysis further showed that the NFκB *cis *element is the motif most strongly associated with aging [64] and that blockade of NFκB in the epidermis of chronologically aged mice resulted in the reversion of both tissue and gene expression characteristics to those of young mice [59]. These and numerous other reports clearly associate increased NFκB activity with multiple aspects of aging including immunosenescence, antigenic stress, innate immunity, tissue atrophy, inflammation, cellular danger responses, apoptosis, DNA damage, oxidative stress and caloric restriction (see [62] for review). Left unchecked, therefore, NFκB activation is a probable driving force for many of these critical aging-related processes.

Given the selectiveness of GTAs in human blood and their novel hydroxylated and unsaturated fatty acid structures, the possibility that human-specific gut microbial processes may be involved in the metabolism of GTAs from dietary sources cannot be excluded. It is also well known that the intestinal microbiome composition changes with age due to a number of contributing factors including reduced mucosal secretion, decreased nutritional status, changes in luminal pH and increased drug and antibiotic use [65-68]. There is also evidence that the gut microbiota is intricately linked to obesity and metabolic syndrome, and can perpetuate insulin-resistance and chronic inflammation [69-71], all of which have been previously implicated with colon cancer [42,72-75]. Lastly, it is intriguing to consider that the modulation of gut microbial composition through the consumption of probiotics and/or fermented milk products has been shown to reduce inflammation and protect against cancer [76-78]. Therefore, investigating the possible interaction between various gut micro-organisms and dietary precursors with respect to GTA metabolism is highly justified.

In summary, our findings collectively suggest a mechanism whereby the age-related decline in GTAs in a subset of the general population results in an impaired ability to control chronic inflammation, which over time may lead to oncogenic cellular changes. The measurement of GTAs may therefore represent an opportunity for the early identification of subjects with elevated inflammatory status and subsequent risk of CRC.

## List of abbreviations

CRC: colorectal cancer; GTA: gastrointestinal tract carboxylic acid; PARP: poly(ADP)-ribose polymerase; ADP: adenosine diphosphate; nuclear factor kappa B; NOS2: nitric oxide synthase 2; IL-1β: interleukin 1 beta; TNFα: tumor necrosis factor alpha; COX2: cyclooxygenase 2; AA: arachidonic acid; DHA: docosehexaenoic acid; EPA: eicosapentanoic acid; TIC: total ion current; TOF: time-of-flight; PUFAs: polyunsaturated fatty acids; IAPs: inhibitor of apoptosis proteins; NIK: NFκB inducible kinase; TLR: Toll-like receptor; PAMP: pathogen-associated molecular pattern; IKKα: IkappaB kinase alpha.

## Competing interests

All authors were employees of Phenomenome Discoveries, Inc. during the course of the work presented in the manuscript. Dayan B. Goodenowe is the president and CEO, and primary shareholder of Phenomenome.

## Authors' contributions

All authors have read and approved the final manuscript. SR: Lead author, wrote the manuscript, directed and oversaw the research presented. DJ: Purification of serum extracts, chromatography, interpretation of MS data, GFD: western blots, PA: lead development of chromatographic methods, interpretation of MS data, WJ: analysis of samples on HPLC-tandem MS, HM: ELISA experiments, nitrite determinations, cell culture and treatments, and DBG: group leader, interpretation and contribution to writing.
